# Patient Preferences for Post‐Radical Cystectomy Treatment in Muscle‐Invasive Bladder Cancer: A Discrete Choice Experiment in Japan

**DOI:** 10.1111/iju.70032

**Published:** 2025-03-10

**Authors:** Shugo Yajima, Shinro Hata, Naoya Masumori, Yoh Matsuoka, Atsuro Sawada, Jun Miki, Mitsuhiro Tambo, Yasuyuki Kobayashi, Ayumu Matsuda, Keita Nakane, Takashi Kobayashi, Hajime Tanaka, Noriya Yamaguchi, Go Kaneko, Russell Miller, Takehiro Seto, Hiroaki Ito, Eiji Kikuchi

**Affiliations:** ^1^ National Cancer Center Hospital East Chiba Japan; ^2^ Oita University Faculty of Medicine Oita Japan; ^3^ Sapporo Medical University School of Medicine Hokkaido Japan; ^4^ Saitama Cancer Center Saitama Japan; ^5^ University of Miyazaki Miyazaki Japan; ^6^ The Jikei University Kashiwa Hospital Chiba Japan; ^7^ Kyorin University School of Medicine Tokyo Japan; ^8^ Okayama University Graduate School of Medicine Okayama Japan; ^9^ National Cancer Center Hospital Tokyo Japan; ^10^ Gifu University Graduate School of Medicine Gifu Japan; ^11^ Kyoto University Graduate School of Medicine Kyoto Japan; ^12^ Institute of Science Tokyo Tokyo Japan; ^13^ Faculty of Medicine Tottori University Tottori Japan; ^14^ Saitama Medical University International Medical Center Saitama Japan; ^15^ Syneos Health Japan K.K. Tokyo Japan; ^16^ Ono Pharmaceutical Co., Ltd. Tokyo Japan; ^17^ Bristol Myers Squibb Tokyo Japan; ^18^ St. Marianna University School of Medicine Kanagawa Japan

**Keywords:** bladder cancer, discrete choice experiment, Japan, patient preference, patient‐centered care

## Abstract

**Objectives:**

This study aimed to assess the treatment preferences of Japanese patients with muscle‐invasive bladder cancer (MIBC) by quantifying their trade‐offs between treatment attributes using a discrete choice experiment (DCE).

**Methods:**

A DCE was conducted among MIBC patients post‐radical cystectomy. Participants were presented with hypothetical treatment options differing in attributes such as efficacy Disease‐free survival (DFS), side effects, administration, and cost. Preference data were analyzed to determine each attribute's relative importance (RI), maximum acceptable risk (MAR), and willingness to trade efficacy (WTTE).

**Results:**

Out of 105 patients surveyed, 101 were included in the final analysis. The DCE revealed that DFS had the highest RI (30.3%), followed by annual treatment cost (27.7%) and risk of severe side effects (26.8%). While MAR analysis indicated that patients were willing to accept some increase in side effects risk for significant gains in DFS, WTTE results showed a sensitivity to side effect severity and treatment costs, with patients often willing to trade off DFS for improvements in risks and costs. In subgroup analyses, such as pathological stage, age, and employment status, the trends for each subgroup were the same as those of the overall population. However, the magnitude of RI varied among patients with different characteristics. For example, younger patients preferred short‐term treatment duration over longer, employed patients tended to favor long‐term treatment duration.

**Conclusions:**

This study is the first to demonstrate the treatment preferences of Japanese MIBC patients who underwent radical cystectomy, and these results can inform treatment selection based on patient preferences.

**Trial Registration:**

Japan Registry of Clinical Trials: jRCT1030220680

## 1 Introduction

Bladder cancer affects 23 185 people annually in Japan, predominantly in individuals aged 60 years or older, with men being three times more likely to develop the disease than women [1]. Bladder cancer is typically categorized into three pathological types: nonmuscle invasive bladder cancer, muscle invasive bladder cancer (MIBC), and metastatic bladder cancer [2, 3]. Approximately 30% of bladder cancers are initially diagnosed as MIBC [3], and approximately 40% of patients develop local recurrence or distant metastasis to other organs within 5 years [4].

The “Clinical Practice Guideline for Bladder Cancer (2019)” by the Japanese Urological Association recommends radical cystectomy (RC) as the standard treatment for MIBC [5], but perioperative chemotherapy is also performed. Cisplatin‐based neoadjuvant chemotherapy (NAC) has been shown to improve overall survival (OS) in several randomized studies [6, 7]. With regard to postoperative chemotherapy in MIBC patients, the EORTC 30994 trial [8] and a study using the National Cancer Database [9] have indicated clinical benefits of cisplatin‐based chemotherapy for pT3‐4 and/or pN+ patients who have not received NAC. Additionally, adjuvant therapy (AT) may also be necessary for patients with ypT2 or higher, even if they have undergone NAC, as they share similar risks of recurrence or metastasis.

As treatment options for MIBC continue to evolve, nivolumab has been approved for AT, offering additional choices for patients. The goal of AT remains the prevention of cancer recurrence, with the selection of anticancer drugs guided by a balance of therapeutic efficacy, side effects, and the patient's lifestyle and social context. In line with the growing emphasis on patient‐centered medicine, it is increasingly important to consider the patient's perspective when deciding on AT. However, there is a lack of research on the factors that MIBC patients prioritize when considering AT after RC, particularly in Japan.

To address this gap, this study aimed to identify the key attributes influencing MIBC patients' treatment preferences for AT. Additionally, we sought to assess the relative importance (RI) of these attributes and explore potential differences in treatment preferences based on varying demographic and clinical characteristics among patients in Japan.

## 2 Methods

The study comprised two phases: Phase 1 involved qualitative interviews to inform the development of a discrete choice experiment (DCE) questionnaire, whereas Phase 2 consisted of a quantitative survey using the finalized questionnaire. Eligible MIBC patients were recruited through physician referrals from approximately 15 sites across Japan (Figure S1).

Phase 1 consisted of a targeted literature review (TLR) and patient interviews to inform the development of a DCE questionnaire. The TLR identified factors influencing MIBC patients' decision‐making regarding post‐RC treatment, such as disease‐free survival (DFS), side effects, dosage frequency, and cost [10, 11, 12, 13, 14, 15], which guided the design of concept elicitation (CE) interviews. These semi‐structured interviews, conducted with 10 patients across six sites, aimed to identify key treatment attributes, refined based on the results of a TLR and expert input. Cognitive debriefing (CD) interviews with five patients subsequently validated these attributes and levels. Appendix S1 provides the rationales for the setting of attributes and levels. Table 1 presents the finalized attributes and levels. All interviews were conducted one‐on‐one in a virtual setting. Phase 2 involved the finalized questionnaire; the allocation was done by block randomization, and patients who met eligibility requirements were asked by physicians at each site to participate in a questionnaire. Patients were given a paper or online option to complete the questionnaire.

**TABLE 1:** Attributes, definitions, and levels included in DCE.

Attribute	Definition	Levels
Disease‐free survival (DFS) (from start of adjuvant therapy)	For the median number of years, patients do not have any cancer after starting adjuvant therapy	1 year, 2 years, 3 years
Probability of serious side effects	Percentage of a grade 3 or higher side effect due to adjuvant therapy	8%, 18%, 72%
Probability of side effects: fatigue	Percentage of patients who experience fatigue due to adjuvant therapy	12%, 17%, 37%
Convenience (frequency and mode of administration)	Route of treatment administration and frequency of administration	Infusions once every week Infusions once every 2 weeks Infusions once every 4 weeks
Treatment duration	Total duration for continuous administration of adjuvant therapy	2 months, 4 months, 1 year
Annual treatment costs	Annual out‐of‐pocket costs for adjuvant therapy	10 000 JPY 300 000 JPY 650 000 JPY

*Note:* While the table refers to “adjuvant treatment” for clinical accuracy, the original DCE questionnaire shared with patients used the term “treatment” to maintain simplicity and avoid technical language.

Although this study did not formally assess concept saturation, the sample size of 10 patients for concept elicitation was based on previous research suggesting that 95% of the most salient concepts are typically identified within the first 10 interviews [16]. In Phase 2, over 100 respondents provided a basis for modeling preference data [17]. Given the relatively rare nature of MIBC and the limited number of post‐RC patients in Japan (including untreated cases), the target sample size for this phase was set at approximately 100 participants.

The study included patients aged 18 years or older, residing in Japan, with a clinical diagnosis of MIBC, able to speak and read Japanese. Eligible patients were those with MIBC pathological staging ypT2–ypT4a or ypN+ who had received NAC and undergone RC, as well as those with pT3–pT4a or pN+ MIBC who had not received NAC but had undergone RC. All participants provided written informed consent.

Patients were excluded if deemed unsuitable by the referring physician. Additional exclusions were patients with other primary cancers requiring treatment at consent, those enrolled in another MIBC clinical trial, patients who experienced recurrence after RC, and (for Phase 1 only) patients receiving or having received adjuvant nivolumab therapy.

Sociodemographic and clinical characteristics were collected in both study phases. In Phase 1, data were obtained from physicians via case report forms (CRFs) and patient interviews; in Phase 2, data were collected from survey participants and physicians via CRFs.

A 100% D‐efficient choice design was constructed using SAS V9.4 M7, generating 36 choice tasks with varying attribute levels. To reduce cognitive burden, tasks were divided into three blocks of 12 tasks each. Design restrictions avoided dominated or implausible tasks, resulting in a final D‐efficiency score of 96.2. One dominant task was added to each block to assess participant rationality. Participants were randomly assigned to one of the three blocks.

Statistical analyses were conducted using SAS V9.4M7. Continuous variables were summarized with descriptive statistics; categorical variables were summarized by frequency and percentage. No imputation was performed for missing data; unanswered questions were coded as missing.

A random parameter logit (RPL) model with effect coding was used to estimate preference weights, RI, maximum acceptable risk (MAR), and willingness to trade efficacy (WTTE). RI was calculated as a percentage based on the difference between the highest and lowest coefficient values.

Using RPL model results, MAR and 95% confidence intervals (CIs) were calculated via the delta method for serious side effects and fatigue, concerning increases in DFS from 1 to 2 years, 2 to 3 years, and 1 to 3 years, using population‐level parameters as per previous methods [18].

DFS was analyzed as a continuous variable to calculate the marginal WTTE for other attributes. WTTE reflects how much an individual is willing to trade for a one‐unit change in an attribute, which was calculated by dividing attribute coefficients by the efficacy coefficient. 95% CIs were calculated using the delta method [19].

Subgroup analyses were conducted based on the following variables: pathological stage, receipt of NAC, age, and employment status to explore potential differences in treatment preferences.

## 3 Results

### 3.1 Demographics

The survey was conducted between March and July 2024, with 105 patients participating. Of these, 101 patients who correctly answered the dominant task were included in the final data analysis. Among them, 77.2% were male, with a median age of 75.0, 80.2% of patients had an ECOG Performance Status of 0, and none had greater than 2 (Table 2). The median time since RC was 15 months. Among the patients, 69.3% had received NAC. Furthermore, 20.8% had a pathological stage of T2N0, and 43.6% received AT following RC. Additionally, 64.8% were unemployed.

**TABLE 2:** Demographic and clinical characteristics of participating patients.

Characteristics (*N* = 101)	*n* (%)
Collected from physicians through CRF	
Sex	
Female	23 (22.8)
Male	78 (77.2)
Age, median (Q1, Q3)	75.0 (69.0, 80.0)
Under 75 years old	50 (49.5)
75 years old and above	51 (50.5)
Frailty score (modified frailty index), mean (SD)	1.09 (1.0)
Modified Charlson comorbidity index category	
0	74 (73.3)
1	18 (17.8)
2	8 (7.9)
3+	1 (1.0)
Functional status (ECOG PS)	
0	81 (80.2)
1	20 (19.8)
Operative method for urinary diversion	
Ileal conduit	74 (73.3)
Cutaneous ureterostomy	21 (20.8)
Orthotopic neobladder	6 (5.9)
Time since MIBC diagnosis (*n* = 100) [month]	
Median (Q1, Q3)	20.5 (10.0, 44.0)
Min, max	3.0, 260.0
Time since RC surgery (*n* = 100) [month]	
Median (Q1, Q3)	15.0 (4.5, 40.0)
Min, max	0.0, 220.0
Less than 2 years	63 (63.0)
Two years or longer	37 (37.0)
Previous pre‐RC treatments for MIBC	
NAC	70 (69.3)
Other	31 (30.7)
No treatment	29 (93.5)
BCG	1 (3.2)
TURBT	1 (3.2)
NAC regimen for MIBC (*n* = 70)	
GC	44 (62.9)
GCarbo	11 (15.7)
ddMVAC	7 (10.0)
Standard MVAC	2 (2.9)
GCarbo + PTX	1 (1.4)
EP	1 (1.4)
Others	4 (5.7)
NAC cycles	
1	3 (4.3)
2	22 (31.4)
3	25 (35.7)
4+	20 (28.6)
Pathological stage on post‐RC	
T2N0	21 (20.8)
Other	80 (79.2)
T3‐4N0	47 (46.6)
TX‐4N1‐3	31 (30.7)
TX‐4NX	2 (2.0)
Received adjuvant therapy post‐RC	
Yes	44 (43.6)
No	57 (56.4)
Necessity of any treatment after RC (physician's decision), (*n* = 57)	
Yes	43 (75.4)
No	14 (24.6)
Physicians have discussed potential need for further treatment post‐RC with their patients (*n* = 57)	
Yes	46 (80.7)
No	11 (19.3)
Time since adjuvant therapy post‐RC (*n* = 28) [months]	
Median (Q1, Q3)	8.5 (2.0, 32.5)
Min, max	0.0, 209.0
Collected from patients through questionnaires	
Education level	
University or graduate school	28 (27.7)
Junior college or technical college	12 (11.9)
High school	49 (48.5)
Junior high school	11 (10.9)
Missing	1 (1.0)
Employment status	
Employed (full‐time)	24 (23.8)
Employed (part‐time)	10 (9.9)
Unemployed	45 (44.6)
Retired	21 (20.8)
Missing	1 (1.0)
Marital status	
Yes	79 (78.2)
No	21 (20.8)
Missing	1 (1.0)
Overall health rating	
Excellent	1 (1.0)
Very good	15 (14.9)
Good	53 (52.5)
Fair	26 (25.7)
Poor	5 (5.0)
Missing	1 (1.0)
Patients had prior discussion with physician regarding potential need for post‐RC treatment	
Yes	72 (71.3)
No	28 (27.7)
Missing	1 (1.0)

Abbreviations: BCG, Bacillus Calmette‐Guerin; CRF, Case Report Form; ddMVAC, dose‐dense Methotrexate + Vinblastine, Doxorubicin, and Cisplatin; ECOG PS, Eastern Cooperative Oncology Group Performance Status; EP, Etoposide + Cisplatin; GC, Gemcitabine + Cisplatin; GCarbo, Gemcitabine + Carboplatin; GCarbo + PTX: Gemcitabine + Carboplatin + Paclitaxel; MIBC, Muscle Invasive Bladder Cancer; MVAC, Methotrexate + Vinblastine, Doxorubicin, and Cisplatin; NAC, Cisplatin‐based Neoadjuvant Chemotherapy; RC, radical cystectomy; TURBT, transurethral resection of the bladder tumor.

### 3.2 Preferences for Attributes and Levels

#### 3.2.1 Relative Importance

**FIGURE 1:**
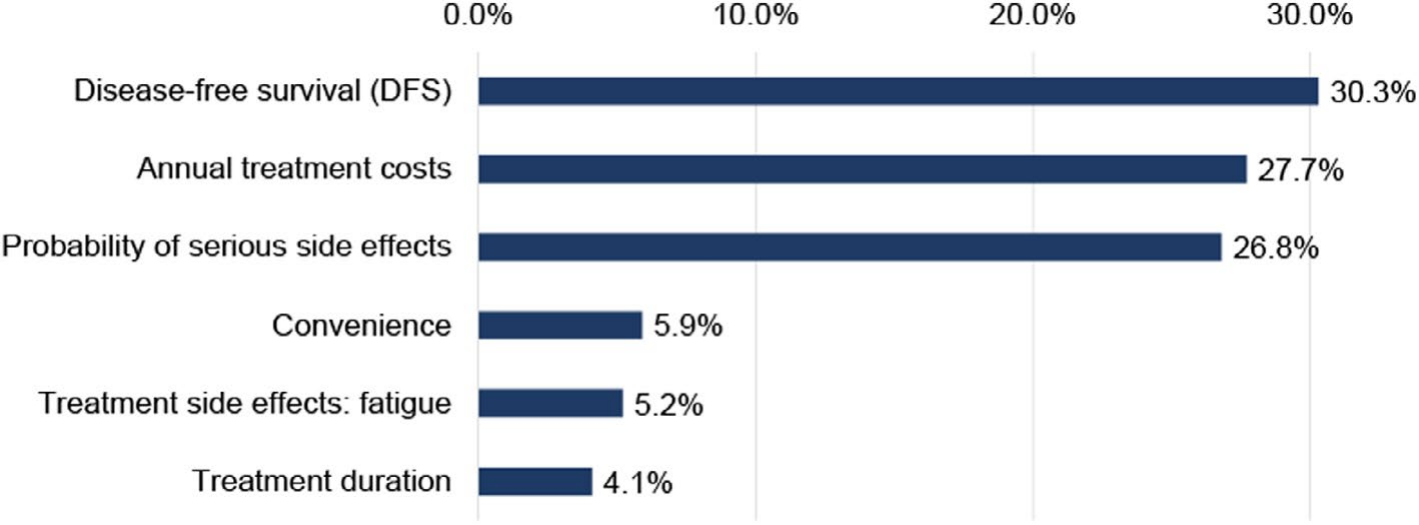
Relative importance.

Across all patients, DFS was the most important attribute (RI: 30.3%), followed by treatment costs (RI: 27.7%) and the probability of serious side effects (RI: 26.8%) (Figure 1). Convenience and fatigue had lower RIs of 5.9% and 5.2%, respectively, with treatment duration being the least important (RI: 4.1%).

#### 3.2.2 RI Across Subgroups Analyses

**FIGURE 2:**
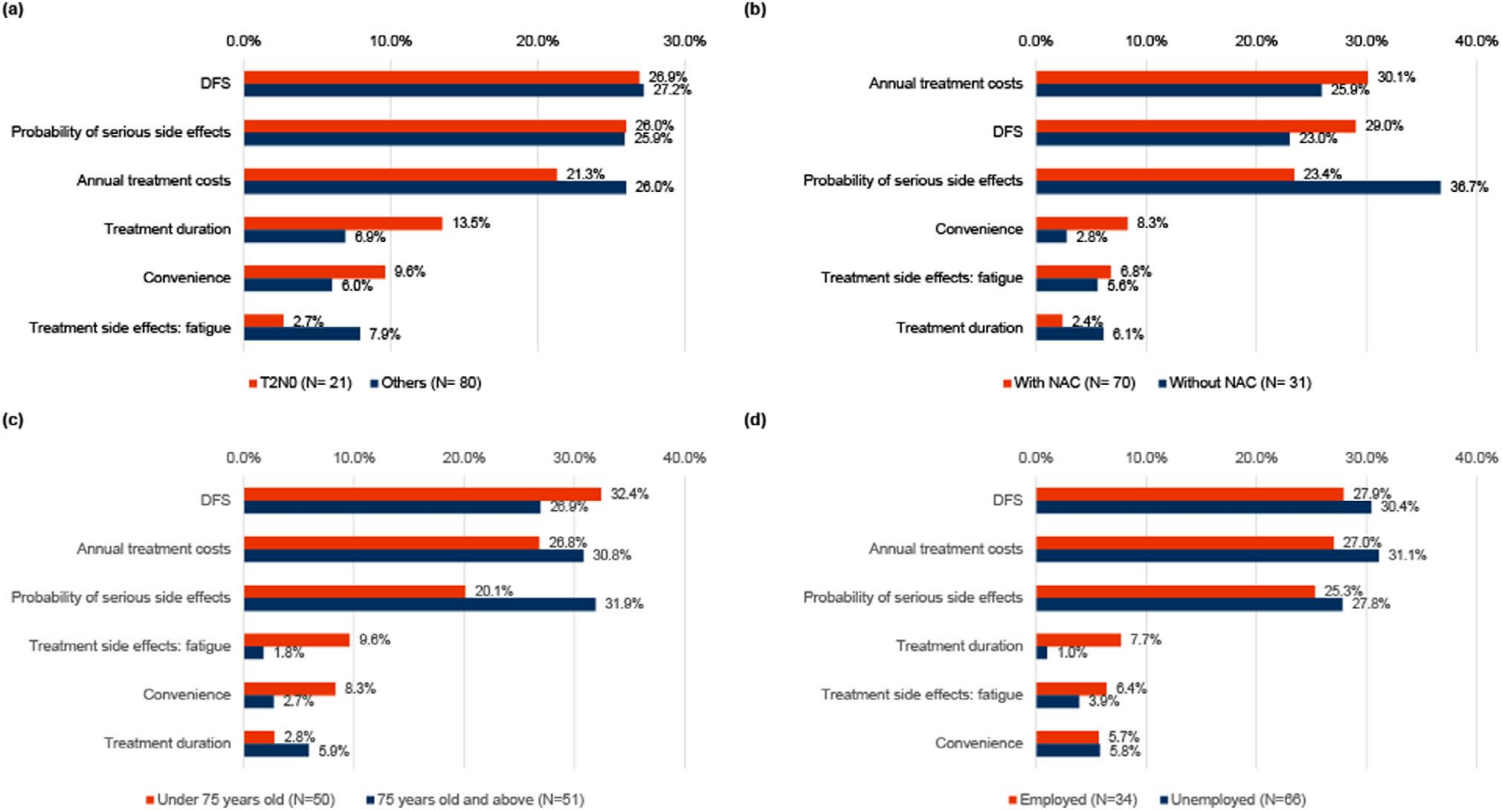
Relative importance of four sub‐group analysis: (a) relative importance by pathological stage, (b) relative importance by the presence of NAC, (c) relative importance by age, (d) relative importance by employment status. DFS, disease‐free survival.

Subgroup analyses (Figure 2) consistently identified DFS, cost, and the probability of serious side effects as key attributes. However, RI varied by group. The trend of the importance of DFS, serious side effects, and costs for patients with T2N0 was similar to that for patients with Others (defined as the group other than T2N0), while there were differences in treatment duration and convenience. Patients without NAC placed greater emphasis on serious side effects (RI: 36.7%) than did those with NAC (RI: 23.4%). Age also influenced preferences, with younger patients (< 75 years) prioritizing DFS, while older patients (≥ 75 years) focused more on reducing serious side effects and convenience. Assessment by employment status further revealed differences. Unemployed patients prioritized treatment cost (RI: 31.1%) and DFS (RI: 30.4%). In contrast, employed patients prioritized DFS (RI: 27.9%) and cost (RI: 27.0%) and were also more interested in treatment duration than unemployed patients.

#### 3.2.3 Preference Weights

**FIGURE 3:**
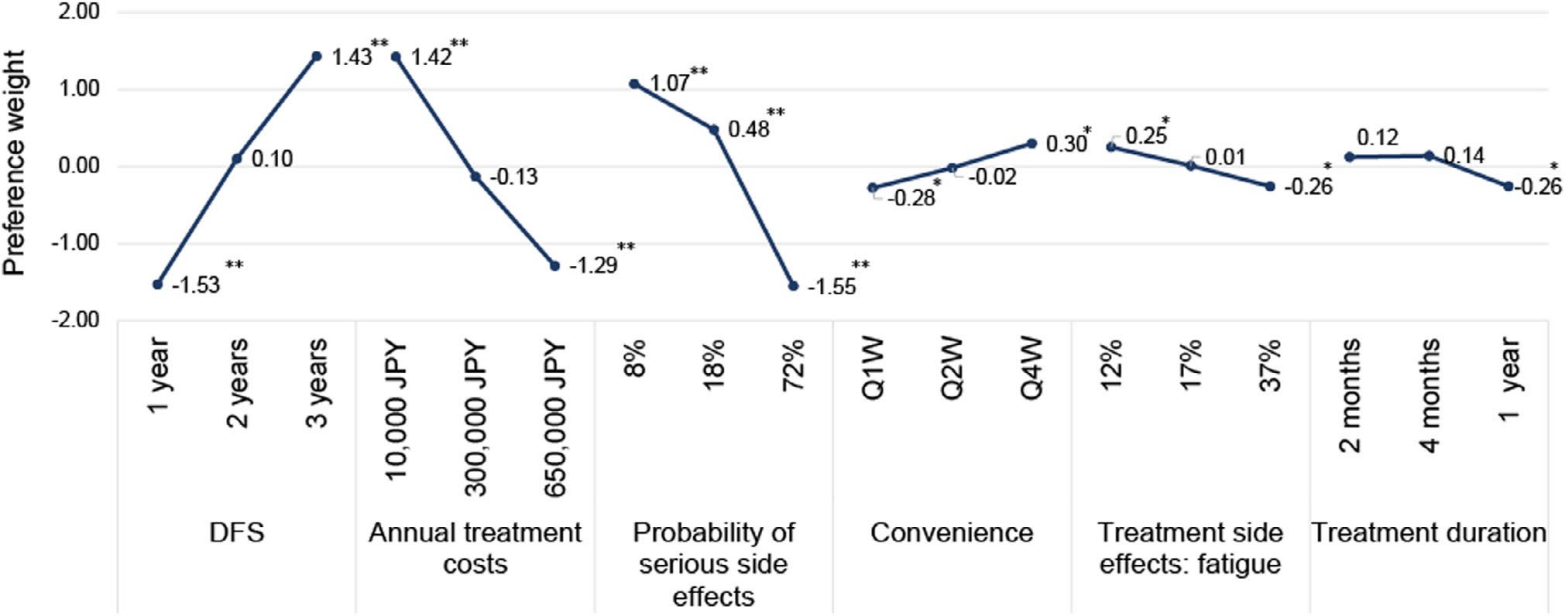
Preference weight. DFS, disease‐free survival, Q1W, once every week; Q2W, once every 2 weeks; Q4W, once every 4 weeks. *p < 0.05, **p < 0.0001.

Figure 3 presents the preference weights from the effects‐coded random logit model. Patients significantly preferred a DFS of 3 years, an annual treatment cost of 10 000 yen, and a probability of serious side effects of 8% or 18% compared to the mean effect of all attribute levels. In contrast, a DFS of 1 year, a probability of serious side effects of 72%, and an annual treatment cost of 650 000 yen were significantly undesirable compared to the mean effect of all attribute levels.

### 3.3 Risk Tolerance and Willingness to Trade

#### 3.3.1 Maximum Acceptable Risk

For MAR (Table 3), patients were willing to accept higher risks of serious side effects and fatigue for larger gains in DFS, though this willingness diminished with smaller improvements, such as moving from 2 to 3 years. Patients were willing to accept a 40% risk of serious side effects to gain an additional year of DFS (from 1 year), with a relatively narrow confidence interval (95% CI: 0.30, 0.50), indicating high confidence in this estimate. This risk tolerance increased to 72% for a 2‐year gain but dropped significantly for the smaller gain from 2 to 3 years. In terms of fatigue as a treatment side effect, patients were willing to tolerate up to 79% for an additional year of DFS, though the wide confidence interval (95% CI: 0.09, 1.49) suggested greater variability in patient preferences. Tolerance similarly decreased when the DFS gain was from 2 to 3 years (95% CI: 0.12, 1.17).

**TABLE 3:** Maximum acceptable risk estimates—effect coded.

Efficacy improvements	From	To	Probability of serious side effects		Probability of side effects: fatigue	
			Mean	95% CI	Mean	95% CI
DFS	1 year	2 years	0.40	0.30, 0.50	0.79	0.09, 1.49
	2 years	3 years	0.33	0.22, 0.43	0.64	0.12, 1.17
	1 year	3 years	0.72	0.59, 0.86	1.43	0.25, 2.62

*Note:* Maximum acceptable risk illustrates how patient risk tolerance varies depending on the magnitude of the improvement in DFS.

Abbreviation: DFS, disease‐free survival.

#### 3.3.2 Willingness to Trade Efficacy

WTTE (Table 4) demonstrated that patients were sensitive to treatment cost and serious side effects. They were willing to trade 0.39 years (95% CI: 0.07, 0.72) of DFS for an 18%–8% decrease in side effects risk and up to 1.92 years (95% CI: 1.15, 2.68) for a 72%–8% decrease. For annual treatment costs, patients would trade 1.09 (95% CI: 0.66, 1.53) to 1.87 years (95% CI: 1.18, 2.56) to avoid 300 000–650 000 JPY increases, indicating strong cost sensitivity. Negative willingness trade‐offs (−0.28 and −0.45 years) indicated that patients were unwilling to trade efficacy for less frequent infusions.

**TABLE 4:** Willingness to trade efficacy for disease‐free survival (unit: year).

Attribute (*N* = 101)	Level	WTTE for DFS (unit: year)	
		Mean	95% CI
Annual treatment costs	10 000 JPY (ref)	—	—
	300 000 JPY	1.09	0.66, 1.53
	650 000 JPY	1.87	1.18, 2.56
Probability of serious side effects	8% (ref)	—	—
	18%	0.39	0.07, 0.72
	72%	1.92	1.15, 2.68
Convenience (frequency and mode of administration)	Once every week (ref)	—	—
	Once every 2 weeks	−0.28	−0.57, 0.01
	Once every 4 weeks	−0.45	−0.74, −0.16
Probability of side effects: fatigue	12% (ref)	—	—
	17%	0.18	−0.06, 0.42
	37%	0.48	0.16, 0.81
Treatment duration	2 months (ref)	—	—
	4 months	0.05	−0.2, 0.3
	1 year	0.23	−0.05, 0.51

*Note:* WTTE indicates the strength of the willingness to choose other attributes even at the expense of treatment effectiveness, representing a trade‐off with DFS.

Abbreviations: DFS, disease‐free survival; WTTE, willingness to trade efficacy.

## 4 Discussion

A key strength of this study for ensuring data quality was conducting a qualitative survey (Phase 1) with 15 patients from six sites. Six treatment attributes and their levels were finalized: DFS, probability of serious side effects, probability of fatigue, frequency of infusions, treatment duration, and annual treatment cost. The interviews revealed that DFS was more important than OS, aligning with findings from the UPRISE study that muscle‐invasive urothelial carcinoma (MIUC) patients had anxiety about the recurrence of tumors at a higher value than OS [20].

Our study highlights that DFS was the most important attribute, with a RI of 30.3%. This result is supported by previous preference studies in urothelial carcinoma (UC) patients [21, 22]. Patients with advanced UC preferred fewer severe side effects than OS, while a study for patients with MIUC who underwent radical resection found that OS is more important than side effects [22]. Efficacy may be more important than safety in the perioperative period, and this result also emphasized efficacy like DFS. DFS is required for MIBC patients because UPRISE also revealed that MIUC patients have anxiety about recurrence [20]. However, some subgroups considered safety important. Patients without NAC prioritized serious side effects. These patients may have concerns about unknown side effects that they have never experienced. Moreover, older patients (≥ 75 years) place greater emphasis on serious side effects and costs than DFS, reflecting differences in values based on lifestyle. Discussions tailored to older patients should include the frequency and impact of AEs, helping them weigh risks and benefits in line with their priorities.

Treatment costs (RI: 27.7%) ranked almost as high as DFS, with many patients willing to trade years of DFS to avoid higher costs. About 70% of patients were unemployed, which may have influenced the overall RI, though we lacked detailed demographic data (e.g., life insurance coverage, standard of living) to fully understand the financial priorities of this group. Unemployed patients may often prioritize financial concerns due to limited income, whereas employed individuals may focus more on balancing treatment, such as treatment duration, with their work responsibilities. A meta‐analysis of cancer patients and survivors, Comprehensive Score for Financial Toxicity (COST) is a key consideration for patients and was significantly linked to a deterioration in health‐related quality of life (HRQOL) [23]. Similarly, a study of Japanese cancer patients suggested that younger age (< 65 years), lack of private health insurance, and lower household income were associated with higher financial toxicity, negatively impacting the quality of life (QOL) [24]. These findings align with our results, suggesting that, even in Japan, unemployed patients and those with limited income may be particularly vulnerable to financial stress regarding cancer treatment. Patients' understanding of financial support systems, including subsidies and insurance, may vary by age and income level. Encouraging consultations with social workers could help patients navigate these systems and mitigate financial toxicity. Agarwal et al. suggested incorporation of multi‐disciplinary team members such as nurses, social workers, counselors, and pharmacists as referral services for patients to discuss costs and financial concerns may assist management [25]. Despite Japan's high‐cost medical expense subsidy program, the significance of cost remains high, implying that financial considerations may have an even greater impact on patient preferences in countries with less robust healthcare systems.

Findings regarding MAR further illuminate patient decision‐making. Patients were generally willing to accept higher risks of serious side effects and fatigue for larger gains in DFS, but this willingness diminished when the survival benefits were marginal. For example, a 40% risk of serious side effects was acceptable for gaining one additional year of DFS and up to a 72% risk for a 2‐year gain; however, for extending from 2 to 3 years, the willingness decreased sharply. This echoes the finding from Fernández et al. [26], where patients were more tolerant of treatment toxicity when larger survival benefits were at stake.

This variability suggests that treatment decisions must be personalized. Turk et al. demonstrated similar patterns in chronic pain patients, who were willing to tolerate more severe side effects in exchange for better pain relief but were less inclined to accept risks for modest improvements [27]. These parallels between MIBC and other therapeutic areas suggest a broader cross‐disease pattern in patient behavior: the larger the perceived benefit, the greater the risk tolerance, but smaller gains do not justify substantial increases in treatment burden or risk. Moreover, our subgroup analyses showed that risk tolerance varies by patient characteristics. In fact, patients without NAC and older patients showed a preference for avoiding serious side effects.

This variability highlights the need for personalized treatment approaches, as different patient characteristics influence treatment preferences. Some patients prioritize cost and side effects, whereas others prioritize survival regardless of the financial or physical burden. This highlights the importance of shared decision‐making, where patients are fully informed of the trade‐offs. Previous research has emphasized the oncologist's role in guiding patients through these complex decisions, particularly in balancing survival outcomes against QOL [26]. The risks and costs of cancer therapy complicate decision‐making in MIBC. Patients' willingness to trade DFS for lower costs or fewer side effects underscores the need to address these concerns. Developing treatment guidelines incorporating patient preferences is also essential to better align recommendations with patient values.

DCE is widely recommended for evaluating patient preferences regarding treatment characteristics due to its superiority in quantitatively capturing complex trade‐offs in decision‐making. However, there may still be gaps between stated and actual preferences [28, 29], as real‐world choices can differ from hypothetical scenarios. Key attributes were selected through a comprehensive literature review, patient interviews, and urologist input to minimize bias. Based on this research, some relevant attributes were not included (e.g., OS). Another limitation is the inherent selection bias, as the study relied on data from patients willing to participate, whose preferences may not fully represent those of the broader MIBC patient population. Furthermore, detailed patient background information, such as the degree of life insurance coverage or living standards in the unemployed group, was not collected, potentially limiting a deeper understanding of how socioeconomic factors influence preferences. While no universal guidelines exist for determining the optimal sample size for DCE studies [30], our sample size aligns with the Japanese MIBC population and was recruited from diverse regions with strict inclusion criteria to ensure meaningful outcomes.

In conclusion, this study underscores DFS as the attribute that most influences the treatment preferences of MIBC patients, followed by annual treatment cost and the probability of serious side effects, while the risk tolerance varies by patient characteristics. This study is the first to show the treatment preferences of Japanese MIBC patients, and these results can inform treatment selection based on patient preferences.

## Author Contributions

**Shugo Yajima:** writing – review and editing, investigation. **Shinro Hata:** investigation, writing – review and editing. **Naoya Masumori:** writing – review and editing, investigation. **Yoh Matsuoka:** writing – review and editing, investigation. **Atsuro Sawada:** writing – review and editing, investigation. **Jun Miki:** investigation, writing – review and editing. **Mitsuhiro Tambo:** investigation, writing – review and editing. **Yasuyuki Kobayashi:** investigation, writing – review and editing. **Ayumu Matsuda:** investigation, writing – review and editing. **Keita Nakane:** investigation, writing – review and editing. **Takashi Kobayashi:** investigation, writing – review and editing. **Hajime Tanaka:** investigation, writing – review and editing. **Noriya Yamaguchi:** investigation, writing – review and editing. **Go Kaneko:** investigation, writing – review and editing. **Russell Miller:** writing – original draft, methodology, formal analysis, data curation, project administration. **Takehiro Seto:** writing – review and editing, supervision, methodology. **Hiroaki Ito:** funding acquisition, writing – review and editing, methodology, supervision. **Eiji Kikuchi:** investigation, writing – review and editing, methodology, supervision, conceptualization.

## Ethics Statement

Before the initiation, the study was approved by a central ethics committee (NPO MINS, approval no, 220225) and independent review committees of each site, and it follows Ethical Guidelines for Medical and Health Research Involving Human Subjects to ensure the protection and rights of all participants.

## Conflicts of Interest

Naoya Masumori has received consulting fees for clinical trials/institutions from Ono Pharmaceutical Co. Ltd. and Bristol Myers Squibb and honoraria for lectures from Ono Pharmaceutical Co. Ltd. Takashi Kobayashi has received research funds from Astellas Pharma Inc., AstraZeneca K.K., Bayer Pharma, and Chugai Pharmaceutical Co. Ltd. and advisory fees from Astellas Pharma Inc., AstraZeneca K.K., Merck Biopharma Co. Ltd., and Chugai Pharmaceutical Co. Ltd., and speakers bureaus from Astellas Pharma Inc., AstraZeneca K.K., Bristol Myers Squibb, Chugai Pharmaceutical Co. Ltd., Merck Biopharma Co. Ltd., and Ono Pharmaceutical Co. Ltd. Go Kaneko has received lecture fees from Bristol Myers Squibb, Ono Pharmaceutical Co. Ltd., Astellas Pharma Inc., Merck Biopharma Co. Ltd., and MSD K.K participated in the Advisory Board of Merck Biopharma Co. Ltd. and MSD K.K. Russell Miller is an employee of Syneos Health Japan. Takehiro Seto is an employee of Ono Pharmaceutical Co. Ltd. Hiroaki Ito is an employee of Bristol Myers Squibb and owns stock or stock options in the company. Eiji Kikuchi has received consulting fees from Astellas Pharma Inc., Bristol Myers Squibb, Janssen, Merck Biopharma Co. Ltd., MSD K.K., Kissei Pharmaceutical Co. Ltd., and Chugai Pharmaceutical Co. Ltd. and lecture fees from Astellas Pharma Inc., Bristol Myers Squibb, Janssen, Merck Biopharma Co. Ltd., MSD K.K., NIPPON KAYAKU Co. Ltd., Taiho Pharmaceutical, Kissei Pharmaceutical Co. Ltd., and Chugai Pharmaceutical Co. Ltd. Naoya Masumori, Yoh Matsuoka, Takashi Kobayashi, Hajime Tanaka, and Eiji Kikuchi are Editorial Board members of the International Journal of Urology and the co‐authors of this article. To minimize bias, they were excluded from all editorial decision‐making related to the acceptance of this article for publication. Except for the above, the authors declare no conflicts of interest.

## Supporting information


**Figure S1.** Study design of the survey.

**Appendix S1.** Rationales for setting attributes and levels.
