# Supplementary figures and images for: Patient Preferences for Post‐Radical Cystectomy Treatment in Muscle‐Invasive Bladder Cancer: A Discrete Choice Experiment in Japan
Source: Int J Urol. 2025 Mar 10;32(6):688–97. doi: 10.1111/iju.70032 (PMC12146242; doi:10.1111/iju.70032)

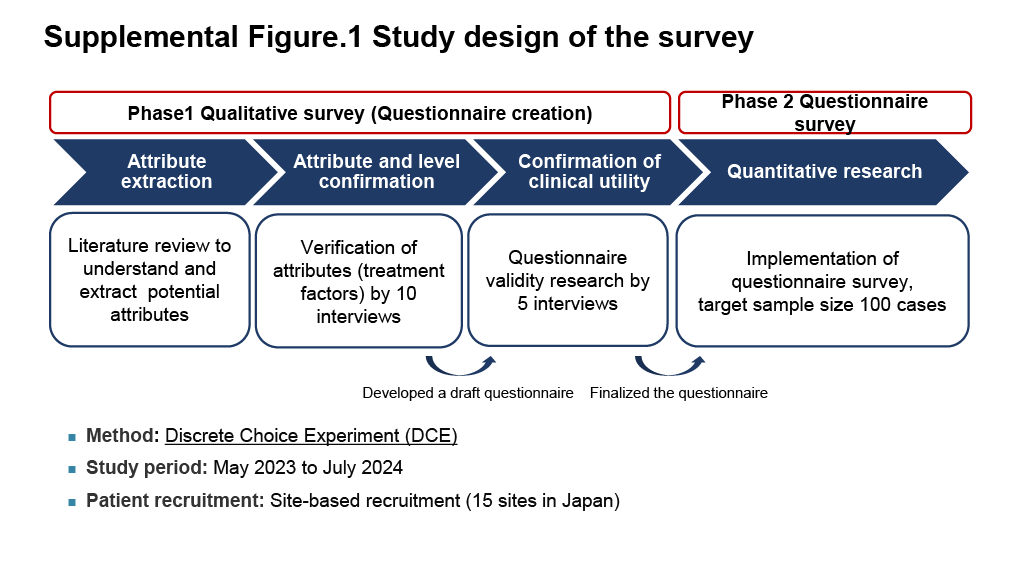

Supplement: Supplementary file 1 — Figure S1. Study design of the survey. [file IJU-32-688-s002.tiff]
