# Supplementary material for: Patient Preferences for Post‐Radical Cystectomy Treatment in Muscle‐Invasive Bladder Cancer: A Discrete Choice Experiment in Japan
Source: Int J Urol. 2025 Mar 10;32(6):688–97. doi: 10.1111/iju.70032 (PMC12146242; doi:10.1111/iju.70032)
Supplement: Supplementary file 2 — Appendix S1. Rationales for setting attributes and levels. [file IJU-32-688-s001.docx]

**Supplemental Document 1. Rationale for setting attributes and levels**

Regarding attributes, key factors influencing treatment selection were identified by combining a literature review, patient interviews, and discussions with a physician. Based on these insights, scenarios for no treatment, chemotherapy, and adjuvant therapy with nivolumab were established, and levels for each attribute were set. Annual treatment costs (②and③) were calculated under the assumption that patients under 70 years old would use Japan’s high-cost medical expense subsidy program (as for patients aged 70 and over without income comparable to the working population, the treatment costs for chemotherapy and adjuvant therapy with nivolumab would not differ significantly, making this level inappropriate).

- **Chemotherapy**: Under the above assumption, drug costs of 80,000 yen/month × 3 months + outpatient costs amount to approximately 300,000 JPY
- **Adjuvant therapy with nivolumab**: Under the above assumption, drug costs of 80,000 yen/month × 3 months + 44,000 yen/month × 9 months amount to approximately 650,000 JPY

| **Attribute** | **Definition** | **Levels** | **Details of rationale/comments** |
| --- | --- | --- | --- |
| Disease-free survival (DFS) (from start of adjuvant therapy) | For the median number of years, patients do not have any cancer after starting adjuvant therapy. | ① 1 year | Assuming no treatment^1^ |
|  |  | ② 2 years | Assuming adjuvant therapy with nivolumab^1^ |
|  |  | ③ 3 years | Assuming chemotherapy^2^ |
| Probability of serious side effects | Percentage of a grade 3 or higher side effect due to adjuvant therapy. | ① 8% | Assuming no treatment^1^ |
|  |  | ② 18% | Assuming adjuvant therapy with nivolumab^1^ |
|  |  | ③ 72% | Assuming chemotherapy^3^ |
| Probability of side effects: fatigue | Percentage of patients who experience fatigue due to adjuvant therapy. | ① 12% | Assuming no treatment^1^ |
|  |  | ② 17% | Assuming adjuvant therapy with nivolumab^1^ |
|  |  | ③ 37% | Assuming chemotherapy^3^ |
| Convenience (frequency and mode of administration) | Route of treatment administration and frequency of administration. | ① Infusions once every week | Assuming chemotherapy |
|  |  | ② Infusions once every 2 weeks | Assuming adjuvant therapy with nivolumab administered at 2-week intervals |
|  |  | ③ Infusions once every 4 weeks | Assuming adjuvant therapy with nivolumab administered at 4-week intervals |
| Treatment duration | Total duration for continuous administration of adjuvant therapy. | ① 2 months | Assuming two cycles of chemotherapy with each cycle lasting one month |
|  |  | ② 4 months | Assuming four cycles of chemotherapy with each cycle lasting one month |
|  |  | ③ 1 year | Assuming adjuvant therapy with nivolumab |
| Annual treatment costs | Annual out-of-pocket costs for adjuvant therapy. | ① 10,000 JPY | Assuming no treatment (regular checkups and hospital visit costs) |
|  |  | ② 300,000 JPY | Assuming chemotherapy |
|  |  | ③ 650,000 JPY | Assuming adjuvant therapy with nivolumab |

**References.** **1.** Bajorin DF, Witjes JA, Gschwend JE, et al. Adjuvant Nivolumab versus Placebo in Muscle-Invasive Urothelial Carcinoma [published correction appears in N Engl J Med. 2021 Aug 26;385(9):864. doi: 10.1056/NEJMx210012]. N Engl J Med. 2021;384(22):2102-2114. doi:10.1056/NEJMoa2034442., **2.** Sternberg CN, Skoneczna I, Kerst JM, et al. Immediate versus deferred chemotherapy after radical cystectomy in patients with pT3-pT4 or N+ M0 urothelial carcinoma of the bladder (EORTC 30994): an intergroup, open-label, randomised phase 3 trial. Lancet Oncol. 2015;16(1):76-86. doi:10.1016/S1470-2045(14)71160-X., **3.** Powles T, Csőszi T, Özgüroğlu M, et al. Pembrolizumab alone or combined with chemotherapy versus chemotherapy as first-line therapy for advanced urothelial carcinoma (KEYNOTE-361): a randomised, open-label, phase 3 trial. Lancet Oncol. 2021;22(7):931-945. doi:10.1016/S1470-2045(21)00152-.
